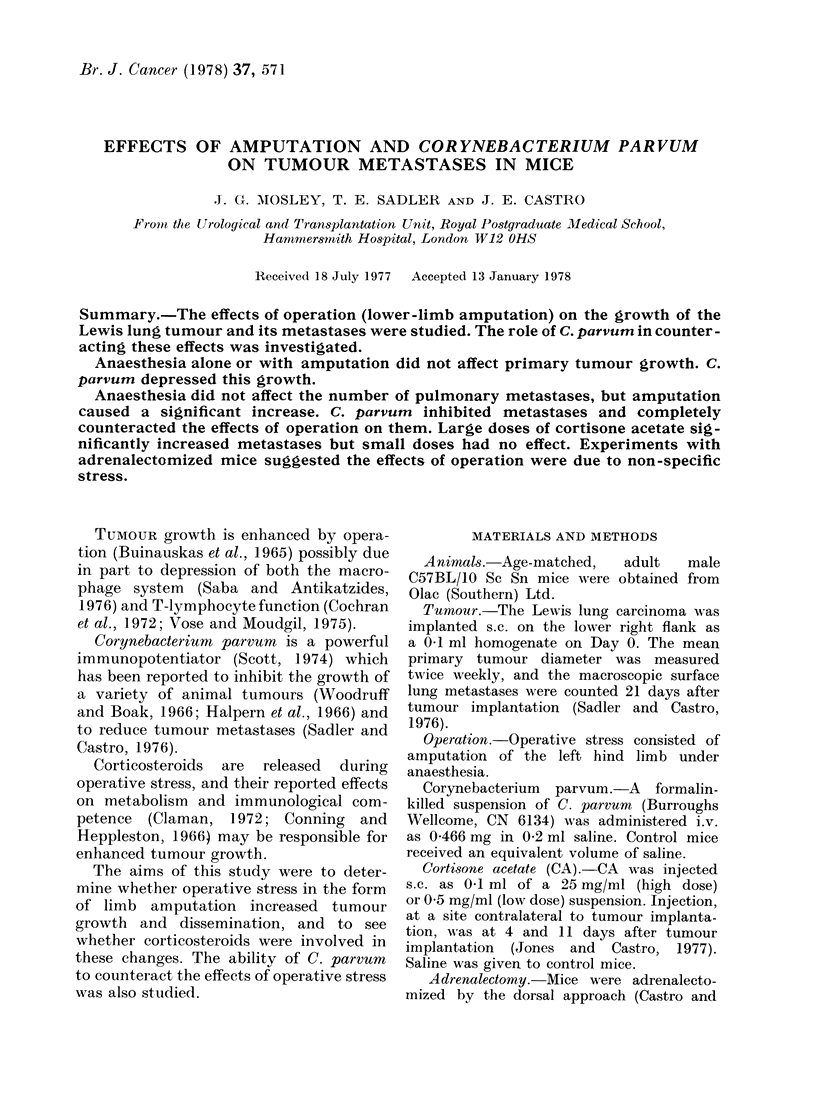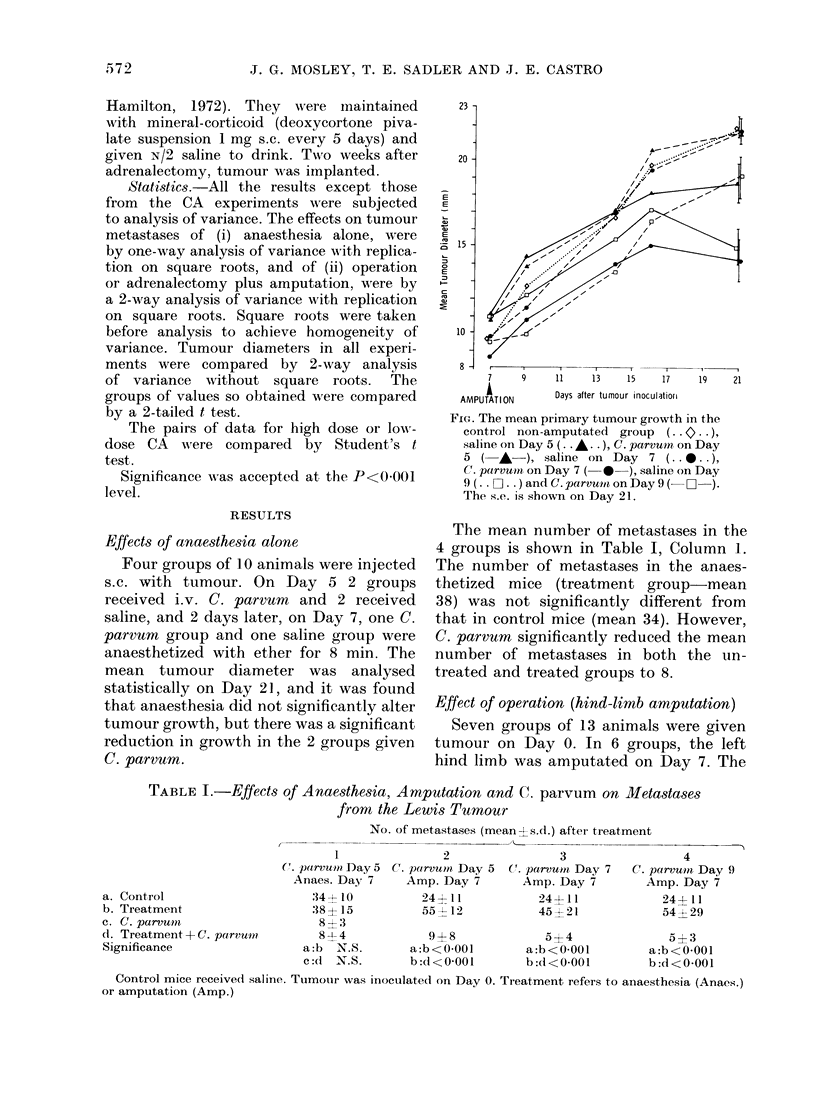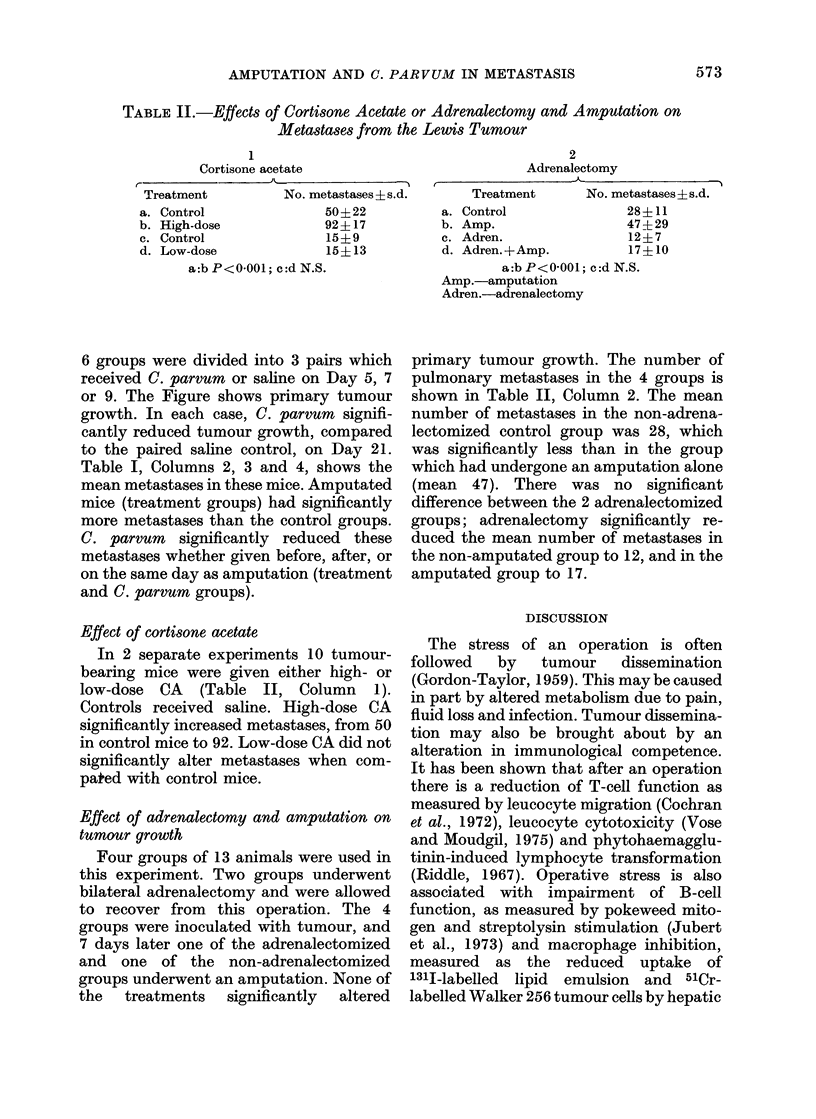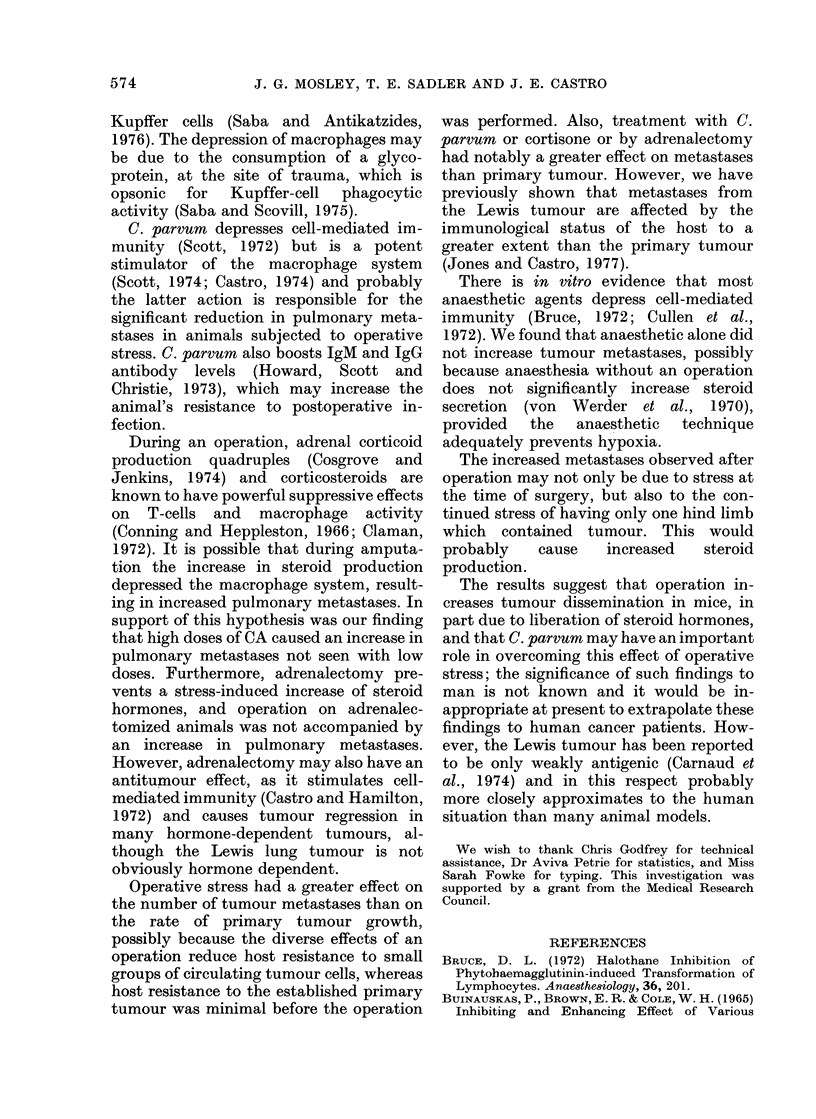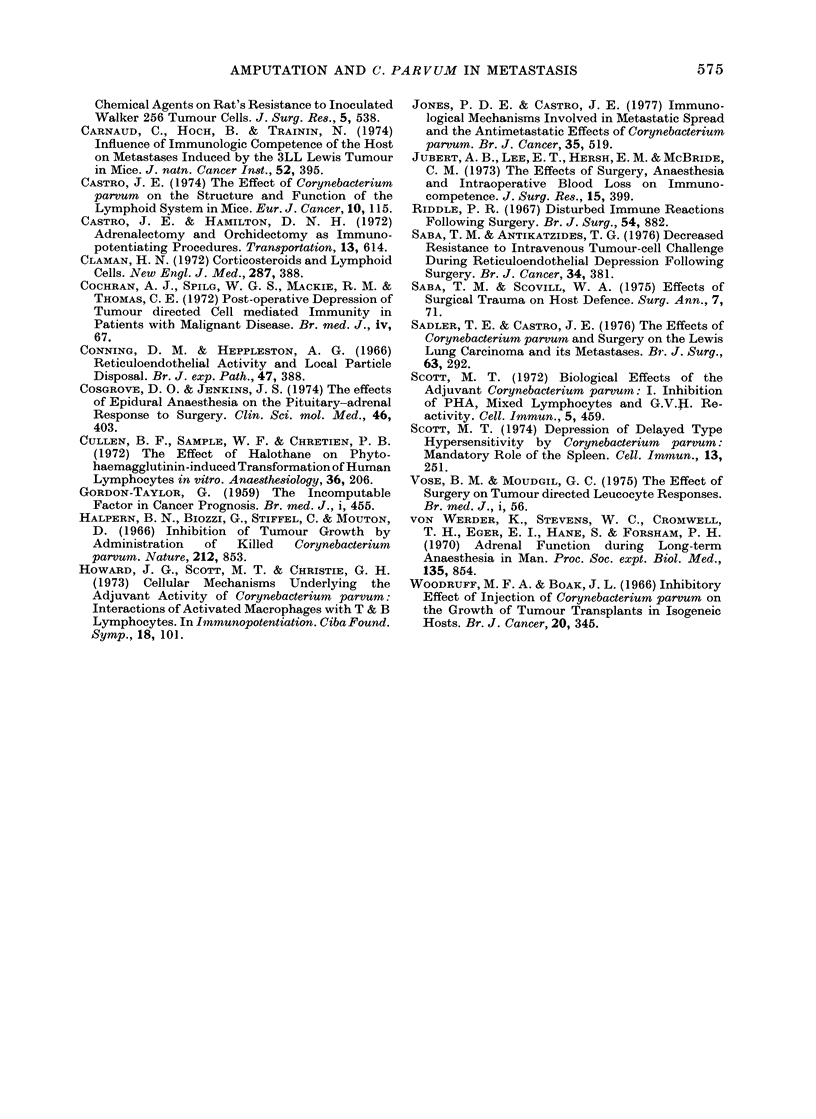# Effects of amputation and Corynebacterium parvum on tumour metastases in mice.

**DOI:** 10.1038/bjc.1978.86

**Published:** 1978-04

**Authors:** J. G. Mosley, T. E. Sadler, J. E. Castro

## Abstract

The effects of operation (lower-limb amputation) on the growth of the Lewis lung tumour and its metastases were studied. The role of C. parvum in counteracting these effects was investigated. Anaesthesia alone or with amputation did not affect primary tumour growth. C. parvum depressed this growth. Anaesthesia did not affect the number of pulmonary metastases, but amputation caused a significant increase. C. parvum inhibited metastases and completely counteracted the effects of operation on them. Large doses of cortisone acetate significantly increased metastases but small doses had no effect. Experiments with adrenalectomized mice suggested the effects of operation were due to non-specific stress.


Br. J. Cancer (1978) 37, 571

### EFFECTS OF AMPUTATION AND *CORYNEBACTERIUM PARVUM* ON TUMOUR METASTASES IN MICE

J. G. MOSLEY, T. E. SADLER AND J. E. CASTRO

*From the Urological and Transplantation Unit, Royal Postgraduate Medical School, Hammersmith Hospital, London W12 0HS*

Received 18 July 1977 Accepted 13 January 1978

**Summary.—The effects of operation (lower-limb amputation) on the growth of the Lewis lung tumour and its metastases were studied. The role of *C. parvum* in counteracting these effects was investigated.**

**Anaesthesia alone or with amputation did not affect primary tumour growth. *C. parvum* depressed this growth.**

**Anaesthesia did not affect the number of pulmonary metastases, but amputation caused a significant increase. *C. parvum* inhibited metastases and completely counteracted the effects of operation on them. Large doses of cortisone acetate significantly increased metastases but small doses had no effect. Experiments with adrenalectomized mice suggested the effects of operation were due to non-specific stress.**

TUMOUR growth is enhanced by operation (Buinauskas *et al.*, 1965) possibly due in part to depression of both the macrophage system (Saba and Antikatzides, 1976) and T-lymphocyte function (Cochran *et al.*, 1972; Vose and Moudgil, 1975).

*Corynebacterium parvum* is a powerful immunopotentiator (Scott, 1974) which has been reported to inhibit the growth of a variety of animal tumours (Woodruff and Boak, 1966; Halpern *et al.*, 1966) and to reduce tumour metastases (Sadler and Castro, 1976).

Corticosteroids are released during operative stress, and their reported effects on metabolism and immunological competence (Claman, 1972; Conning and Heppleston, 1966) may be responsible for enhanced tumour growth.

The aims of this study were to determine whether operative stress in the form of limb amputation increased tumour growth and dissemination, and to see whether corticosteroids were involved in these changes. The ability of *C. parvum* to counteract the effects of operative stress was also studied.

### MATERIALS AND METHODS

*Animals.*—Age-matched, adult male C57BL/10 Sc Sn mice were obtained from Olac (Southern) Ltd.

*Tumour.*—The Lewis lung carcinoma was implanted s.c. on the lower right flank as a 0·1 ml homogenate on Day 0. The mean primary tumour diameter was measured twice weekly, and the macroscopic surface lung metastases were counted 21 days after tumour implantation (Sadler and Castro, 1976).

*Operation.*—Operative stress consisted of amputation of the left hind limb under anaesthesia.

Corynebacterium parvum.—A formalin-killed suspension of *C. parvum* (Burroughs Wellcome, CN 6134) was administered i.v. as 0·466 mg in 0·2 ml saline. Control mice received an equivalent volume of saline.

*Cortisone acetate* (CA).—CA was injected s.c. as 0·1 ml of a 25 mg/ml (high dose) or 0·5 mg/ml (low dose) suspension. Injection, at a site contralateral to tumour implantation, was at 4 and 11 days after tumour implantation (Jones and Castro, 1977). Saline was given to control mice.

*Adrenalectomy.*—Mice were adrenalectomized by the dorsal approach (Castro and

Hamilton, 1972). They were maintained with mineral-corticoid (deoxycortone pivalate suspension 1 mg s.c. every 5 days) and given N/2 saline to drink. Two weeks after adrenalectomy, tumour was implanted.

*Statistics.*—All the results except those from the CA experiments were subjected to analysis of variance. The effects on tumour metastases of (i) anaesthesia alone, were by one-way analysis of variance with replication on square roots, and of (ii) operation or adrenalectomy plus amputation, were by a 2-way analysis of variance with replication on square roots. Square roots were taken before analysis to achieve homogeneity of variance. Tumour diameters in all experiments were compared by 2-way analysis of variance without square roots. The groups of values so obtained were compared by a 2-tailed $t$ test.

The pairs of data for high dose or low-dose CA were compared by Student's $t$ test.

Significance was accepted at the $P<0{\cdot}001$ level.

### RESULTS

### *Effects of anaesthesia alone*

Four groups of 10 animals were injected s.c. with tumour. On Day 5 2 groups received i.v. *C. parvum* and 2 received saline, and 2 days later, on Day 7, one *C. parvum* group and one saline group were anaesthetized with ether for 8 min. The mean tumour diameter was analysed statistically on Day 21, and it was found that anaesthesia did not significantly alter tumour growth, but there was a significant reduction in growth in the 2 groups given *C. parvum*.

FIG. The mean primary tumour growth in the control non-amputated group (..◇..), saline on Day 5 (..▲..), *C. parvum* on Day 5 (—▲—), saline on Day 7 (..●..), *C. parvum* on Day 7 (—●—), saline on Day 9 (..□..) and *C. parvum* on Day 9 (—□—). The s.e. is shown on Day 21.

The mean number of metastases in the 4 groups is shown in Table I, Column 1. The number of metastases in the anaesthetized mice (treatment group—mean 38) was not significantly different from that in control mice (mean 34). However, *C. parvum* significantly reduced the mean number of metastases in both the untreated and treated groups to 8.

### *Effect of operation (hind-limb amputation)*

Seven groups of 13 animals were given tumour on Day 0. In 6 groups, the left hind limb was amputated on Day 7. The

TABLE I.—*Effects of Anaesthesia, Amputation and* C. parvum *on Metastases from the Lewis Tumour*

| | No. of metastases (mean ± s.d.) after treatment | | | |
|---|---|---|---|---|
| | 1 *C. parvum* Day 5 Anaes. Day 7 | 2 *C. parvum* Day 5 Amp. Day 7 | 3 *C. parvum* Day 7 Amp. Day 7 | 4 *C. parvum* Day 9 Amp. Day 7 |
| a. Control | 34 ± 10 | 24 ± 11 | 24 ± 11 | 24 ± 11 |
| b. Treatment | 38 ± 15 | 55 ± 12 | 45 ± 21 | 54 ± 29 |
| c. *C. parvum* | 8 ± 3 | | | |
| d. Treatment + *C. parvum* | 8 ± 4 | 9 ± 8 | 5 ± 4 | 5 ± 3 |
| Significance | a:b N.S. | a:b < 0·001 | a:b < 0·001 | a:b < 0·001 |
| | c:d N.S. | b:d < 0·001 | b:d < 0·001 | b:d < 0·001 |

Control mice received saline. Tumour was inoculated on Day 0. Treatment refers to anaesthesia (Anaes.) or amputation (Amp.)

TABLE II.—*Effects of Cortisone Acetate or Adrenalectomy and Amputation on Metastases from the Lewis Tumour*

| 1 Cortisone acetate | | 2 Adrenalectomy | |
|---|---|---|---|
| Treatment | No. metastases ± s.d. | Treatment | No. metastases ± s.d. |
| a. Control | 50±22 | a. Control | 28±11 |
| b. High-dose | 92±17 | b. Amp. | 47±29 |
| c. Control | 15±9 | c. Adren. | 12±7 |
| d. Low-dose | 15±13 | d. Adren.+Amp. | 17±10 |
| a:b $P<0\cdot001$; c:d N.S. | | a:b $P<0\cdot001$; c:d N.S. | |

Amp.—amputation
Adren.—adrenalectomy

6 groups were divided into 3 pairs which received *C. parvum* or saline on Day 5, 7 or 9. The Figure shows primary tumour growth. In each case, *C. parvum* significantly reduced tumour growth, compared to the paired saline control, on Day 21. Table I, Columns 2, 3 and 4, shows the mean metastases in these mice. Amputated mice (treatment groups) had significantly more metastases than the control groups. *C. parvum* significantly reduced these metastases whether given before, after, or on the same day as amputation (treatment and *C. parvum* groups).

*Effect of cortisone acetate*

In 2 separate experiments 10 tumour-bearing mice were given either high- or low-dose CA (Table II, Column 1). Controls received saline. High-dose CA significantly increased metastases, from 50 in control mice to 92. Low-dose CA did not significantly alter metastases when compared with control mice.

*Effect of adrenalectomy and amputation on tumour growth*

Four groups of 13 animals were used in this experiment. Two groups underwent bilateral adrenalectomy and were allowed to recover from this operation. The 4 groups were inoculated with tumour, and 7 days later one of the adrenalectomized and one of the non-adrenalectomized groups underwent an amputation. None of the treatments significantly altered primary tumour growth. The number of pulmonary metastases in the 4 groups is shown in Table II, Column 2. The mean number of metastases in the non-adrenalectomized control group was 28, which was significantly less than in the group which had undergone an amputation alone (mean 47). There was no significant difference between the 2 adrenalectomized groups; adrenalectomy significantly reduced the mean number of metastases in the non-amputated group to 12, and in the amputated group to 17.

DISCUSSION

The stress of an operation is often followed by tumour dissemination (Gordon-Taylor, 1959). This may be caused in part by altered metabolism due to pain, fluid loss and infection. Tumour dissemination may also be brought about by an alteration in immunological competence. It has been shown that after an operation there is a reduction of T-cell function as measured by leucocyte migration (Cochran *et al.*, 1972), leucocyte cytotoxicity (Vose and Moudgil, 1975) and phytohaemagglutinin-induced lymphocyte transformation (Riddle, 1967). Operative stress is also associated with impairment of B-cell function, as measured by pokeweed mitogen and streptolysin stimulation (Jubert et al., 1973) and macrophage inhibition, measured as the reduced uptake of $^{131}$I-labelled lipid emulsion and $^{51}$Cr-labelled Walker 256 tumour cells by hepatic

Kupffer cells (Saba and Antikatzides, 1976). The depression of macrophages may be due to the consumption of a glycoprotein, at the site of trauma, which is opsonic for Kupffer-cell phagocytic activity (Saba and Scovill, 1975).

*C. parvum* depresses cell-mediated immunity (Scott, 1972) but is a potent stimulator of the macrophage system (Scott, 1974; Castro, 1974) and probably the latter action is responsible for the significant reduction in pulmonary metastases in animals subjected to operative stress. *C. parvum* also boosts IgM and IgG antibody levels (Howard, Scott and Christie, 1973), which may increase the animal's resistance to postoperative infection.

During an operation, adrenal corticoid production quadruples (Cosgrove and Jenkins, 1974) and corticosteroids are known to have powerful suppressive effects on T-cells and macrophage activity (Conning and Heppleston, 1966; Claman, 1972). It is possible that during amputation the increase in steroid production depressed the macrophage system, resulting in increased pulmonary metastases. In support of this hypothesis was our finding that high doses of CA caused an increase in pulmonary metastases not seen with low doses. Furthermore, adrenalectomy prevents a stress-induced increase of steroid hormones, and operation on adrenalectomized animals was not accompanied by an increase in pulmonary metastases. However, adrenalectomy may also have an antitumour effect, as it stimulates cell-mediated immunity (Castro and Hamilton, 1972) and causes tumour regression in many hormone-dependent tumours, although the Lewis lung tumour is not obviously hormone dependent.

Operative stress had a greater effect on the number of tumour metastases than on the rate of primary tumour growth, possibly because the diverse effects of an operation reduce host resistance to small groups of circulating tumour cells, whereas host resistance to the established primary tumour was minimal before the operation was performed. Also, treatment with *C. parvum* or cortisone or by adrenalectomy had notably a greater effect on metastases than primary tumour. However, we have previously shown that metastases from the Lewis tumour are affected by the immunological status of the host to a greater extent than the primary tumour (Jones and Castro, 1977).

There is *in vitro* evidence that most anaesthetic agents depress cell-mediated immunity (Bruce, 1972; Cullen *et al.*, 1972). We found that anaesthetic alone did not increase tumour metastases, possibly because anaesthesia without an operation does not significantly increase steroid secretion (von Werder *et al.*, 1970), provided the anaesthetic technique adequately prevents hypoxia.

The increased metastases observed after operation may not only be due to stress at the time of surgery, but also to the continued stress of having only one hind limb which contained tumour. This would probably cause increased steroid production.

The results suggest that operation increases tumour dissemination in mice, in part due to liberation of steroid hormones, and that *C. parvum* may have an important role in overcoming this effect of operative stress; the significance of such findings to man is not known and it would be inappropriate at present to extrapolate these findings to human cancer patients. However, the Lewis tumour has been reported to be only weakly antigenic (Carnaud *et al.*, 1974) and in this respect probably more closely approximates to the human situation than many animal models.

We wish to thank Chris Godfrey for technical assistance, Dr Aviva Petrie for statistics, and Miss Sarah Fowke for typing. This investigation was supported by a grant from the Medical Research Council.

### REFERENCES

BRUCE, D. L. (1972) Halothane Inhibition of Phytohaemagglutinin-induced Transformation of Lymphocytes. *Anaesthesiology*, **36**, 201.

BUINAUSKAS, P., BROWN, E. R. & COLE, W. H. (1965) Inhibiting and Enhancing Effect of Various

Chemical Agents on Rat's Resistance to Inoculated Walker 256 Tumour Cells. *J. Surg. Res.*, **5**, 538.

CARNAUD, C., HOCH, B. & TRAININ, N. (1974) Influence of Immunologic Competence of the Host on Metastases Induced by the 3LL Lewis Tumour in Mice. *J. natn. Cancer Inst.*, **52**, 395.

CASTRO, J. E. (1974) The Effect of *Corynebacterium parvum* on the Structure and Function of the Lymphoid System in Mice. *Eur. J. Cancer*, **10**, 115.

CASTRO, J. E. & HAMILTON, D. N. H. (1972) Adrenalectomy and Orchidectomy as Immunopotentiating Procedures. *Transportation*, **13**, 614.

CLAMAN, H. N. (1972) Corticosteroids and Lymphoid Cells. *New Engl. J. Med.*, **287**, 388.

COCHRAN, A. J., SPILG, W. G. S., MACKIE, R. M. & THOMAS, C. E. (1972) Post-operative Depression of Tumour directed Cell mediated Immunity in Patients with Malignant Disease. *Br. med. J.*, **iv**, 67.

CONNING, D. M. & HEPPLESTON, A. G. (1966) Reticuloendothelial Activity and Local Particle Disposal. *Br. J. exp. Path.*, **47**, 388.

COSGROVE, D. O. & JENKINS, J. S. (1974) The effects of Epidural Anaesthesia on the Pituitary–adrenal Response to Surgery. *Clin. Sci. mol. Med.*, **46**, 403.

CULLEN, B. F., SAMPLE, W. F. & CHRETIEN, P. B. (1972) The Effect of Halothane on Phytohaemagglutinin-induced Transformation of Human Lymphocytes *in vitro*. *Anaesthesiology*, **36**, 206.

GORDON-TAYLOR, G. (1959) The Incomputable Factor in Cancer Prognosis. *Br. med. J.*, i, 455.

HALPERN, B. N., BIOZZI, G., STIFFEL, C. & MOUTON, D. (1966) Inhibition of Tumour Growth by Administration of Killed *Corynebacterium parvum*. *Nature*, **212**, 853.

HOWARD, J. G., SCOTT, M. T. & CHRISTIE, G. H. (1973) Cellular Mechanisms Underlying the Adjuvant Activity of *Corynebacterium parvum:* Interactions of Activated Macrophages with T & B Lymphocytes. In *Immunopotentiation. Ciba Found. Symp.*, **18**, 101.

JONES, P. D. E. & CASTRO, J. E. (1977) Immunological Mechanisms Involved in Metastatic Spread and the Antimetastatic Effects of *Corynebacterium parvum*. *Br. J. Cancer*, **35**, 519.

JUBERT, A. B., LEE, E. T., HERSH, E. M. & MCBRIDE, C. M. (1973) The Effects of Surgery, Anaesthesia and Intraoperative Blood Loss on Immunocompetence. *J. Surg. Res.*, **15**, 399.

RIDDLE, P. R. (1967) Disturbed Immune Reactions Following Surgery. *Br. J. Surg.*, **54**, 882.

SABA, T. M. & ANTIKATZIDES, T. G. (1976) Decreased Resistance to Intravenous Tumour-cell Challenge During Reticuloendothelial Depression Following Surgery. *Br. J. Cancer*, **34**, 381.

SABA, T. M. & SCOVILL, W. A. (1975) Effects of Surgical Trauma on Host Defence. *Surg. Ann.*, **7**, 71.

SADLER, T. E. & CASTRO, J. E. (1976) The Effects of *Corynebacterium parvum* and Surgery on the Lewis Lung Carcinoma and its Metastases. *Br. J. Surg.*, **63**, 292.

SCOTT, M. T. (1972) Biological Effects of the Adjuvant *Corynebacterium parvum:* I. Inhibition of PHA, Mixed Lymphocytes and G.V.H. Reactivity. *Cell. Immun.*, **5**, 459.

SCOTT, M. T. (1974) Depression of Delayed Type Hypersensitivity by *Corynebacterium parvum:* Mandatory Role of the Spleen. *Cell. Immun.*, **13**, 251.

VOSE, B. M. & MOUDGIL, G. C. (1975) The Effect of Surgery on Tumour directed Leucocyte Responses. *Br. med. J.*, i, 56.

VON WERDER, K., STEVENS, W. C., CROMWELL, T. H., EGER, E. I., HANE, S. & FORSHAM, P. H. (1970) Adrenal Function during Long-term Anaesthesia in Man. *Proc. Soc. expt. Biol. Med.*, **135**, 854.

WOODRUFF, M. F. A. & BOAK, J. L. (1966) Inhibitory Effect of Injection of *Corynebacterium parvum* on the Growth of Tumour Transplants in Isogeneic Hosts. *Br. J. Cancer*, **20**, 345.